# Diagnosis of Barmah Forest Virus Infection by a Nested Real-Time SYBR Green RT-PCR Assay

**DOI:** 10.1371/journal.pone.0065197

**Published:** 2013-07-23

**Authors:** Linda Hueston, Cheryl S. Toi, Neisha Jeoffreys, Tania Sorrell, Gwendolyn Gilbert

**Affiliations:** 1 Centre for Infectious Diseases and Microbiology Laboratory Services, Westmead, NSW, Australia; 2 Sydney Institute for Emerging Infectious Diseases and Biosecurity, Westmead, NSW, Australia; University of Hong Kong, Hong Kong

## Abstract

Barmah Forest virus (BFV) is a mosquito borne (+) ssRNA alphavirus found only in Australia. It causes rash, myalgia and arthralgia in humans and is usually diagnosed serologically. We developed a real-time PCR assay to detect BFV in an effort to improve diagnosis early in the course of infection. The limit of detection was 16 genome equivalents with a specificity of 100%. Fifty five serum samples from BFV-infected patients were tested by the PCR. 52 of 53 antibody-positive samples were PCR negative. Two culture-positive (neutralizing antibody negative) samples were positive on first round PCR, while one sample (IgM and neutralizing antibody strongly positive, IgG negative) was positive on second round PCR, suggesting that viral RNA is detectable and transiently present in early infection. PCR can provide results faster than culture, is capable of high throughput and by sequencing the PCR product strain variants can be characterized.

## Introduction

Barmah Forest virus (BFV) was first isolated in 1974 [1] and is the second most commonly diagnosed arboviral disease in Australia. It was first associated with human disease in 1986 [2]. Symptoms of BFV disease include rash, myalgia, arthralgia and fever [3]. Disease occurrence is seasonal, with the highest incidence in summer and autumn for most of Australia, although spring appears to be the peak season for south-west Western Australia [4]. Diagnosis is usually achieved serologically, largely by the detection of IgM antibody in single samples. However, the persistence of IgM antibody following infection and difficulties with the specificity of some serologic tests has led to inaccuracies in diagnosis and reporting of cases [5]. Detection of nucleic acid with its high specificity and sensitivity would be a useful adjunct to serologic diagnosis helping to overcome some of these issues. We describe the development of a highly sensitive and specific nested RT-PCR for the detection of BFV that can be performed in <8 hrs hrs with high throughput.

## Methods

### Virus strains and isolation

Virus was produced by propagation on BHK-21 cell monolayers in MEM Medium (Gibco Laboratories, USA) containing 1% foetal calf serum until cytopathic effect was evident (approximately 36 hrs post-inoculation). Cell debris was removed by centrifugation at 3000 rpm for 10 minutes. The clarified supernatant was used as virus stock. Two BFV isolates were used, the prototype strain BFV BH2193 and an isolate from the Forster region of NSW obtained from mosquito pools collected during the 1995 outbreak. To determine the specificity of the PCR, five non-BFV isolates were also tested, including one Sindbis, one Getah, one Whataroa, two Chikungunya and two Ross River virus isolates (Table 1). To determine limit of detection, purified whole virions of the prototype strain BFV BH2193 were prepared by ultracentrifugation of clarified tissue culture supernatant and purification of virus particles by sucrose density gradient.

**Table 1:** Specificity of the BFV nested Real-Time RT- qPCR assay.

Alphavirus	RNA concentration	Calculated	Result
	µg/µl	geq/µg	geq/reaction
Ross River Virus T48	56.39	8.14×10^9^	Negative
Ross River Virus 2156	64.61	9.54×10^9^	Negative
Chikungunya (NT)	72.33	1.06×10^10^	Negative
Chikungunya (Mauritius)	35.21	5.17×10^9^	Negative
Sindbis (Leeton)	50.04	7.15×10^9^	Negative
Getah (C636)	46.08	7.02×10^9^	Negative
Whataroa (1337)	76.76	1.19×10^10^	Negative
BFV (Forster)	63.26	9.85×10^9^	8.5×10^8^

### Clinical Samples

Fifty-five serum samples were screened for BFV RNA to evaluate the diagnostic worth and clinical applicability of the assay. The clinical specimens had been submitted for testing, from patients with suspected BFV infection, during an outbreak in 1995 or during the summer of 2009/2010. The samples from 1995 had been stored at −70°C for 15 years while the samples from 2009/2010 had been stored at −20°C. Samples were chosen on the basis of a clinical presentation consistent with BFV infection, virus isolation results and/or serology results. Two of the samples from 1995 had no detectable neutralising antibody but were culture- positive for BFV; 18 samples were IgM- and neutralising antibody-positive but IgG negative and 5 samples were IgG-, IgM- and neutralising antibody-positive. The remaining samples were from 2009/2010 and were culture negative but IgG and IgM antibody positive for BFV. All samples were antibody negative for Ross River virus and Sindbis virus antibody. Ethics approval was obtained through the Human Research Ethics Committee, Western Sydney Local Health District.

### Selection of BFV gene targets

The prototype BFV BH2193 [6] (Genbank: U73745.1) sequence was retrieved from the GenBank database and used as a template for designing oligonucleotide primers using Primer-BLAST (NCBI). Two sets of primers spanning part of the non-structural protein 4, and E2 genes (Table 2) were used to amplify the outer 242-bp (BFVE2f, BFVE2r) and nested 138 bp regions (BFVf2, BFVr2) (Sigma-Aldrich, Castle Hill, NSW, Australia). This region of the BFV genome was chosen because it had a high homology to known BFV sequences, high annealing temperatures and low homology to other alphavirus sequences in GenBank.

**Table 2:** Oligonucleotide primer sequences used in the nested RT-PCR assay.

	Primer	Primer sequence (5′- 3′)	Amplicon size (bp)	Nucleotide position (BFV BH2193)*
Outer	BFVE2f	ACCCATGAGCAAGACAATCC	242	9222–9241
	BFVE2r	GTTCGAGCGACTGAACACAA		9464–9445
Inner	BFVf2	AAGGCGTGGAGTATGTCTGG	138	9254–9273
	BFVr2	CTGTGATGGTCCAGTAAGGG		9391–9372

* Genbank Accession number U73745.1.

### Extraction of viral RNA

Virus RNA was extracted by use of the High Pure Viral RNA Kit (Roche, Mannheim, Germany). Briefly, 200 µl of the purified seed virus, virus culture supernatant and serum samples were extracted according to manufacturer's instruction and eluted in nuclease free, sterile double distilled water in volumes of 100 µl and 50 µl. The concentration of the RNA extract was determined on a Beckman DU® Series 500 spectrophotometer (Beckman, CA, USA) at an absorbance of 260 nm (A_260_) and 280 nm (A_280_) in 10 mM Tris-Cl at pH 7.5. The ratio between the two readings (A_260_/A_280_) provided an estimate of the purity of RNA. The extracts were immediately stored at −80°C until further use.

### Reverse transcription and first round Amplification

Reverse transcription and first-round PCR using the Access RT-PCR System (Promega, Madison, USA) was carried out on the Applied Biosystems Veriti™ thermal cycler (Life Technologies, Carlsbad, USA). Gene specific primers BFVE2f and BFVE2r were used to synthesise first strand cDNA and for second strand DNA amplification of the 242 bp gene target. Briefly, 10 µl of the extracted RNA was added to the RT-PCR reaction mix containing 0.2 µM of the outer primers (BFVE2f and BFVE2r), 200 nM dNTPs, 2 mM MgSO_4_, 0.10 U of avian myoblastoma virus (AMV) reverse transcriptase and Tfl (Thermus flavus) DNA polymerase, 1× AMV/Tfl reaction buffer and nuclease-free water to a final reaction volume of 25 µl. The thermal cycle programme for first strand cDNA synthesis was 45°C for 45 minutes, followed by 94°C for 2 minutes. Second strand cDNA synthesis and PCR amplification consisted of denaturation (20 s at 94°C), annealing (30 s at 68°C), and extension (30 s at 72°C) for 40 cycles. After 10 cycles, the extension cycle was increased by 5 s per cycle for 30 cycles. A final extension was held at 72°C for 5 mins.

### Nested real-time qPCR amplification

A nested, real-time qPCR assay was performed on the LightCycler® 480 (Roche, Mannheim, Germany) system by means of the SYBR Green Master I kit (Roche, Mannheim, Germany). Two µl of the first round amplified product was added to 10 µl of 1× SybrGreen Master Mix, 0.2 µl (0.2 µM) of oligonucleotide primers, BFVf2 and BFVr2 and 7.6 µl PCR grade water to a final volume of 20 µl. The 96-well plate was briefly centrifuged prior to thermal cycling which consisted of incubation at 95°C for 10 mins, followed by 42 cycles of denaturation (10 s at 95°C), annealing (10 s at 68°C) and extension (15 s at 72°C). The acquisition mode was set to acquire fluorescence on the extension cycle followed by a melt curve with ramping temperatures between 65°C–95°C. A sample was considered positive if the quantification cycle (Cq value) was <40 based on background cross-reactivity of the primers in the non-template control. A no-reverse transcription control and non-template controls (NTC) were added to both the RT-PCR and nested qPCR to distinguish non-specific amplification products. NTCs with C_q_s≥40 were ignored when the C_q_ for the lowest concentration unknown was 38. Between 5 µl–8 µl of amplified gene target on both rounds of PCR amplification were checked for product purity and for non-specific priming by electrophoresis on a 2% agarose gel containing SYBR® Safe DNA gel stain (Invitrogen Corporation, Carlsbad, CA, USA).

### Assay evaluation

#### Amplification efficiency

Real-time PCR amplification efficiency was determined by a calibration curve to evaluate the reproducibility, analytical sensitivity and robustness of the assay. A quantification calibrator was constructed with BFV cDNA cloned into plasmids. Barmah Forest purified whole virions (Batch 140903) RNA was reversed transcribed to cDNA with SuperScript™ III Reverse Transcriptase (Invitrogen Corporation, Carlsbad, CA, USA). Ten µl of virus RNA was added to a 20-µl reaction volume containing 2.2 µl sterile distilled water, 1 µl of DTT (0.1 M), 1 µl dNTPs (10 mM), BFVf2 gene specific primer (25 nM), 1× RT Buffer, 0.4 µl RNasin® Plus RNase Inhibitor (Promega Corporation, Madison, WI, USA) and 0.4 µl of SuperScript™ III RT (200 U/µl). The nested region (138 bp) was amplified from BFV (Batch 140903) and 2 µl cloned into the pGEM®-T Easy vector according to the manufacturer's instruction (Promega Corporation, Madison, USA). The plasmid inserts were confirmed by PCR amplification of the target, followed by gel electrophoresis to check that the target was intact, and the insert DNA sequenced.

Plasmid DNA of transformed *Escherichia coli* (JM109) cells were grown in SOC medium and incubated at 37°C overnight with shaking (225 rpm). Thereafter, the DNA was extracted with the PureYield™ Plasmid Midiprep System (Promega Corporation, Madison, WI, USA), and eluted to a final volume of 1500 µl in nuclease-free water and the concentration measured by spectrophotometry. The number of copies was calculated based on the concentration and size of the plasmid (3015 bp) and insert (138 bp) [7]. The standard curve was constructed by ten-fold serial dilutions ranging from 8 to 8.0×10^5^ copies per 5 µl.

### Specificity and Limit of detection

The specificity of the oligonucleotide primer set; BFVE2f and BFVE2r was tested against a panel of alphaviruses that had been previously isolated in culture from mosquito pools and/or clinical specimens. They included Ross River (T48 and 2156 strains), Chikungunya (NT strain- isolated from a traveller from Indonesia and MT strain – isolated from a traveller from Mauritius), Sindbis (Leeton strain), Whataroa (1337 strain) and Getah (C636 strain) viruses (Table 1). In this experiment, Barmah Forest virus (Forster) was included as a positive reference and sterile, nuclease free water as a negative control. The viral RNA was isolated from the culture supernatants of Chikungunya, Whataroa, Getah and BFV (Forster) together with Ross River and Sindbis purified virus. The amount of viral RNA was measured spectrophometrically and the number of RNA molecules calculated. Ten microlitres of each virus RNA containing between 35.21 and 76.76 ng/µl was used for the RT-PCR per reaction applying the PCR conditions described previously to amplify the 242-bp region. This procedure was followed by nested qPCR.

The analytical sensitivity of the assay was determined by relating the PCR signal to input copy number using the plasmid cDNA calibration curve. The minimum number of copies in a sample that could be accurately measured with 95% certainty was considered the limit of detection. Purified BFV (Batch 140903) was extracted and the RNA concentration determined by spectrophotometry. The number of RNA molecules was calculated based on the RNA concentration, genome length of 11486 bp and an average weight of 336.6 g/mol for each nucleotide, yielding a weight of 7.66×10^−9^ ng per RNA molecule. Ten µl of each RNA serial dilution ranging from 0.077–7.7×10^9^ molecules per µl were reverse transcribed and PCR amplified as previously described using the outer primers. The amplicons were verified on a 2% agarose gel and checked for non-specific banding. Nested, real-time qPCR was performed on the highest virus serial dilution in spiked serum which showed a discrete band, as well as the serial dilutions where no banding was evident on gel electrophoresis.

To determine the diagnostic value and sensitivity of the assay, serum from a healthy BFV antibody- negative individual was spiked with a series of 10-fold dilutions of BFV RNA(Batch 140903) ranging from 0.077 to 7.7×10^8^ copies per µl. Viral RNA was extracted from 200 µl of each spike and eluted into a final volume of 50 µl. The concentration of extracted viral RNA in the spiked sera was measured and tested using the methods previously described.

### DNA sequencing and analysis

PCR amplicons were pre-treated with ExoSAP-IT® (USB Corporation, Cleveland, Ohio, USA). Five µl of the PCR product was added to 2 µl of ExoSAP-IT (Exonuclease I and Shrimp Alkaline Phosphatase), and 28 µl of sterile distilled water. The mixture was treated for 15 min at 37°C, followed by inactivation for 15 min at 80°C and held at 22°C in a thermal cycler. A total volume of 12 µl containing 10.8 µl of purified product and 1.2 µl of forward primer (16 pM/µl) were sequenced on a 3730xl DNA Sequencer (Applied Biosystems, Forster City, USA) using the BigDye® Terminator chemistry Version 3.1. The positive clinical samples were confirmed by sequencing and compared against the prototype BFV BH2193 [6] (Genbank: U73745.1). DNA sequences were checked in the BioEdit version 7.0.4.1 [8] sequencing editing program and aligned with ClustalW Multiple alignment [9]. Phylogenetic comparison was made with MEGA Version 4 [10] The Kumura 2-parameter [11] model was used to assess the support for the major clades and phylogenetic trees constructed with the neighbour joining method [12] with bootstrap [13]–[14] resampling (1000 repeats).

### Analysis of data

The sensitivity of the assay was determined by the absolute quantification of test samples extrapolated from the plasmid standard curve using the second derivative maximum method. The dynamic range of the calibration curve extended to 6 log_10_ concentrations. PCR assays with an amplification efficiency of ≥1.9 and standard curve slopes between −3.1 and −3.6 were accepted, and quantification cycle values of test samples >40 were not included in the analysis. The Repeated Measures Analysis of Variance (ANOVA) was used to test inter and intra-experiment variability in six separate experiments on cDNA cloned plasmids, and in four separate experiments on the spiked sera. The accuracy and repeatability of the PCR method was assessed by comparing the measured viral copies of the plasmid clones and the spiked sera, to the calculated copy number.

## Results

### Specificity

The specificity of the RT-PCR, nested- qPCR assay is shown in Table 1. No PCR amplification of either the 242-bp or the nested 138-bp region was evident for the five non-BFV alphaviruses tested, when compared against a BFV negative serum sample and a PCR non-BFV template control. Barmah Forest (Forster) virus alone was detected at a copy number of 8.5×10^8^ molecules of RNA on nested qPCR.

### Limit of detection

The limit of detection of the RT-PCR, using the outer oligonucleotide primers, was 3.85×10^4^ genome equivalents/reaction. This 10^−5^ virus dilution spike contained 18.48 pg of RNA and the amplified 242-bp gene target was visualised on a 2% agarose gel containing SYBR® Safe DNA gel stain. Amplified product from the higher virus dilutions between 10^−6^ and 10^−14^ was not visible on the agarose gel. Consequently, the sensitivity of the assay on nested, real-time qPCR was enhanced 10000-fold (Table 3) using the inner primers (138 bp). The limit of detection was 16 BFV genome equivalents amplified in the 10^−9^ virus dilution spike.

**Table 3:** Limit of Detection of the BFV real-time qPCR nested assay.

Sample Dilution	Calculated geq	Quantification cycle (C_q_)	Assay Result
	concentration	Mean ± SD	Mean ± SD
cDNA Plasmid Standard	geq/5 µl	N = 6	geq/5 µl
10^−1^	8.0×10^5^	18.52±2.01	8.05×10^5^±5.72×10^4^
10^−2^	8.0×10^4^	22.37±1.88	7.50×10^4^±1.46×10^4^
10^−3^	8.0×10^3^	26.12±1.90	7.42×10^3^±2.50×10^3^
10^−4^	8.0×10^2^	29.47±1.72	9.35×10^2^±3.27×10^2^
10^−5^	8.0×10^1^	33.71±1.96	8.37×10^1^±5.85×10^1^
10^−6^	8.0×10^0^	37.06±2.18	1.18×10^1^±7.16×10^0^

Spiked sera	geq/µl	N = 4*	geq/reaction
10^−5^	2.9×10^4^	24.28±2.28	1.88×10^4^±3.72×10^3^
10^−6^	2.9×10^3^	26.79±2.26	3.98×10^3^±6.77×10^2^
10^−7^	2.9×10^2^	30.52±2.16	3.75×10^2^±4.29×10^1^
10^−8^	2.9×10^1^	34.09±1.32	2.74×10^1^±1.33×10^1^
10^−9^	2.9	35.29±1.01	1.62×10^1^±1.10×10^1^

* P = 0.0033, F = 8.1.

### Reproducibility and Accuracy

A repeated-measures analysis of variance test showed no statistically significant difference in viral genome equivalents and C_q_ between test samples or between experiments for the cDNA plasmid standards. Equally, the numbers of BFV genome equivalents in spiked sera were similar, but a significant difference in C_q_ values (P = 0.0033; F = 8.1) was apparent. Tukey's multiple comparison test showed that the main difference to be between mean C_q_ values of experiment 1 and 4 (P<0.01).

### Clinical samples

A total of 55 human serum samples were screened, three of which were PCR positive for BFV. The C_q_ value and copy number of the positive test samples were; 9108812 (23.43; 5.08×10^5^), S15A (8.81; 1.29×10^5^) and S20A (24.26; 2.89×10^5^). Samples S15A and S20A had been collected in a BFV outbreak in 1995, and cultured positive in BHK-21 and C6/36 cell lines but were neutralising antibody negative. Sample 9108812 was collected in 2010 from a patient with a clinical history of rash and myalgia who had a strongly positive IgM, low level IgG and strong positive neutralising antibody levels. Comparison between the BFV-positive clinical samples and the prototype BFV BH2193, showed that CS20A and CS15A were similar, but 9108812 appeared more closely related to BH2193.

## Discussion

We describe the development of a nested PCR for the rapid detection of BFV in clinical samples. Testing of the BFV-specific primers against five non-BFV alphaviruses showed that the PCR assay was 100% specific for the detection of BFV with no evidence of cross-reactivity using either sets of oligonucleotide primers and the pre-determined thermal cycling conditions. It was therefore not considered necessary to use probe hybridisation techniques.

Slopes between −3.1 and-3.6 giving reaction efficiencies between 90–110% are typically acceptable on the LightCycler® 480.

Sensitivity is often reported in terms of plaque forming units (pfu) but it is difficult to estimate how many virions form 1 pfu. Sellner [15] estimated that one Vero cell pfu may contain 100–1000 virions but noted virion number is affected by cell type used. To overcome this problem we determined sensitivity by the absolute quantification of test samples extrapolated from the plasmid standard curve. Using this method we determined the limit of detection to be 16 genome equivalents.

Investigations undertaken during the 1995 outbreak in NSW suggested that the viraemia in BFV infection in humans is short-lived, based on the observation that virus could not be cultured from samples with detectable neutralising antibody (Hueston, unpublished data). Interestingly the PCR-positive sample from 2010 was culture negative, contained high levels of IgM and neutralising antibody but low levels of IgG antibody, consistent with early infection. This suggests that the PCR is capable of detecting virus, at least early in the course of infection. The fact that it was only positive on the second round PCR suggests that the virus was present at a low level. This indicates that the period during which infection can be detected by RT-PCR, is longer than tissue culture allows. It is unlikely that PCR will replace serology as the mainstay of BFV diagnostics as evidenced by the 52 PCR negative clinically suspected and serologically confirmed cases – but it will be a useful adjunct in diagnosing early BFV infection. Sequencing the PCR product from positive samples will allow investigation of strain variation which may provide useful epidemiologic insights into this uniquely Australian alphavirus.
